# Intelligent career planning via stochastic subsampling reinforcement learning

**DOI:** 10.1038/s41598-022-11872-8

**Published:** 2022-05-18

**Authors:** Pengzhan Guo, Keli Xiao, Zeyang Ye, Hengshu Zhu, Wei Zhu

**Affiliations:** 1 Duke Kunshan University, Kunshan, Jiangsu China; 2 Stony Brook University, Stony Brook, NY USA; 3 Samsung Research America, Mountain View, CA USA; 4 Baidu Talent Intelligence Center, Beijing, China

**Keywords:** Applied mathematics, Computational science, Computer science, Engineering

## Abstract

Career planning consists of a series of decisions that will significantly impact one’s life. However, current recommendation systems have serious limitations, including the lack of effective artificial intelligence algorithms for long-term career planning, and the lack of efficient reinforcement learning (RL) methods for dynamic systems. To improve the long-term recommendation, this work proposes an intelligent sequential career planning system featuring a career path rating mechanism and a new RL method coined as the stochastic subsampling reinforcement learning (SSRL) framework. After proving the effectiveness of this new recommendation system theoretically, we evaluate it computationally by gauging it against several benchmarks under different scenarios representing different user preferences in career planning. Numerical results have demonstrated that our system is superior to other benchmarks in locating promising optimal career paths for users in long-term planning. Case studies have further revealed that our SSRL career path recommendation system would encourage people to gradually improve their career paths to maximize long-term benefits. Moreover, we have shown that the initial state (i.e., the first job) can have a significant impact, positively or negatively, on one’s career, while in the long-term view, a carefully planned career path following our recommendation system may mitigate the negative impact of a lackluster beginning in one’s career life.

## Introduction

A well-planned career mobility strategy exploring both short-term and long-term horizons is essential for individual career success, and beneficial to employers as well as the entire human society, collectively. From the perspective of individuals, having a clear career plan will improve their job performance and fulfilment in life^1,2^. As to the benefits for companies, foreseeing the optimal career paths of employees can facilitate decision making related to promotion, training, assessment, and retainment^3^, hence strengthen a company’s competitiveness and survival^4^. In terms of social benefit, the collective career path trend serves as an important measure of the economic equilibrium in the labor market and projects the supply and demand of knowledge investments^5^. Good estimation and forecasting of career path changes would help bridge the market demand and employee skills^6^, and further resolve the labor shortage as well as the unemployment issues. In summary, optimized career mobility will help reduce social costs associated with job matching, relocation, and other aspects in people’s career and life, and lead to an improved social efficiency and stability.

When making decisions for career mobility, people appraise the opportunity system with job openings, resources, position types, and experience learning^7^. However, human decisions are usually non-optimal due to limited access to data and insufficient information processing ability^6^. Based on statistics from the U.S. Bureau of Labor Statistics (BLS) and the U.S. Census Bureau, from 2010 to 2020, the underemployment rate for college graduates is about 30% in the United States; while the rate increases to 40–50% for recent graduates. Here, underemployment is defined as a condition that people are employed in jobs that are less than full-time positions or at jobs that are mismatched with their training and qualifications. Also, it has been found that people tend to try different jobs to understand their own capacities and interests at the early stage of their careers^8^. Based on the 2019 U.S. BLS survey, the median staying duration in a company is only 3 years for young employee, which is also revealed in our data (Supplementary Fig. S.1). To evaluate career paths, we propose a quality evaluation model that considers both company-related and position-related factors. According to the evaluation model and the data from a well-known professional social network site, real-world career paths receive an average score of merely 41.96 out of 100 points (Supplementary Fig. S.1).

Given the strengths of artificial intelligence (AI) in supporting human decisions, data-driven techniques enable us to address challenging talent analytical tasks in an intelligent and quantitative way, e.g., job-salary benchmarking^9,10^, job recommendation^11–13^, and talent assessment^5^. In this work, we focus on developing novel AI strategies for long-term career planning, an unexplored task in talent analytics. Leveraging AI and stochastic optimization techniques, this work aims to develop an intelligent career planning system geared to provide optimal career path recommendations while heeding personal preferences.

Prior studies of intelligent career planning and long-term recommendation have failed to address three important issues as follows. First, the career planning problem has not been carefully defined in a generalized form. Related work mainly focus on addressing some specific forecasting problems, such as job mobility prediction^12^, person-organization fit^14^, and turnover prediction^15^. There is no well-defined evaluation function to assess career path quality with a long-term perspective. Second, most of the existing models were designed to handle one-step prediction or matching tasks, while career planning is often considered as a long-term task with sage decisions leading to lifetime benefits. Lastly, although reinforcement learning (RL) has found success in long-term strategic planning^16–18^, existing RL algorithms are not efficient enough to handle scenarios with indefinite (i.e., unobservable and unpredictable) and time-varying actions. The career path recommendation task is under exactly such a scenario, given the number of companies is unpredictable and changes over time. Therefore, an efficient algorithm for RL should be developed to deal with the complexity caused by indefinite actions.

Along this line, our work aims to address all the above issues, leading to innovations in both career path planning and the methodology of RL. Specifically, we propose a stochastic subsampling reinforcement learning (SSRL) framework that is capable of locating the globally optimal path on the indefinite set ${\mathscr{C}}$ by exploring on the finite subset ${\mathscr{C}}_{sub}$. SSRL utilizes the subset ${\mathscr{C}}_{sub}$ based on the locally optimal to accelerate the convergence rate and applies an efficient Markov Chain method and the Boltzmann distribution to accelerate the convergence rate towards the global optimum. Using the career path quality evaluation model proposed in this work, without considering specific user preference, our results show that the new methodology achieves an average career path score of 64.78, that is a 54.3% improvement compared with the average score of 41.96 based on real career trajectories. Also, the superiority of the SSRL framework is confirmed under three other scenarios with different user preference settings. With case studies, we demonstrate how our career planning system offers guidance to people for a steady improvement of their career path.

The innovations of this work are threefold. First and foremost, this is the first work addressing the career planning problem as a long-term career path optimization task. We formulate the objective function and propose a rating system for companies, which is customizable based on people’s needs and preferences. Second, to address the career path optimization task, we propose an enhanced variant of RL method with a stochastic subsampling mechanism for path searching. The effectiveness and efficiency of our method are confirmed both theoretically and empirically. Last, in addition to the overall performance check, we provide case studies to demonstrate and compare career recommendations based on different approaches.

## Stochastic subsampling reinforcement learning for career planning

Let ${\mathscr{C}}$ be a collection of companies and ${\mathscr{P}}$ be a collection of all career paths. We define $P_i$ as the *i*th career path in ${\mathscr{P}}$, which contains a sequence of company, job and time period pairs:

1 $$\begin{aligned} P_i = \left\{ \left( C_{1}, J_1, D_{1} \right) , \left( C_{2}, J_2, D_{2} \right) ,\ldots , \left( C_{n}, J_n, D_{n} \right) \right\} , \end{aligned}$$

where $1, 2,\ldots ,n$ are the index sequence indicating the order; $C_{n}$ is a company in $\mathcal{C}$ and on the path $P_i$ with index *n*; $J_n \text{ and } D_{n}$ represent the job and staying duration at $C_{n}$, respectively. Note that the complete forms of *J*_*n*_ and *D*_*n*_ are *J*_*Cn*_ and *D*_*Cn*_. To simplify the notations, we define *J*_*n*_ = *J*_*Cn*_ and *D*_*n*_ = *D*_*Cn*_. For example, the career path in Eq. (1) suggests that a person works in company $C_{1}$ with a job $J_1$ and a staying duration of $D_{1}$, and then move to company $C_{2}$ with job $J_2$ and stay there for $D_{2}$, and so forth.

To evaluate the quality of a given career path, we denote the reward for staying at company $C_i$ by $S_{C_i}$. The objective of our work is to locate the optimal career path $P^*$, which can be defined as:

2 $$\begin{aligned} P^* = \underset{P_i \in \mathscr{P}}{\mathrm{argmax}} \sum _{C_j \in P_i} S_{C_j} \end{aligned}$$

That is, we aim to optimize people’s career path by recommending a sequence of companies and corresponding staying durations, which will result in the highest accumulative reward to an individual. We refer to this task as the *career sequential recommendation* (CSR) problem.

### SSRL framework

Given the strength of RL in sequence planning (e.g., playing Go^16^, protein structure prediction^19^, etc.), we propose a stochastic subsampling reinforcement learning (SSRL) framework to address the above CSR problem. The framework is capable of handling different requirements of career planning by employing the RL and stochastic modeling techniques. The newly proposed stochastic subsampling mechanism not only boosts the path search, but also avoids the use of neural network, which is an important component in traditional deep RL models, hence increases the transparency of the training process of our method.

The framework is formed as a four-step iterating system to handle different requirements of recommendation tasks.

**Figure 1:**
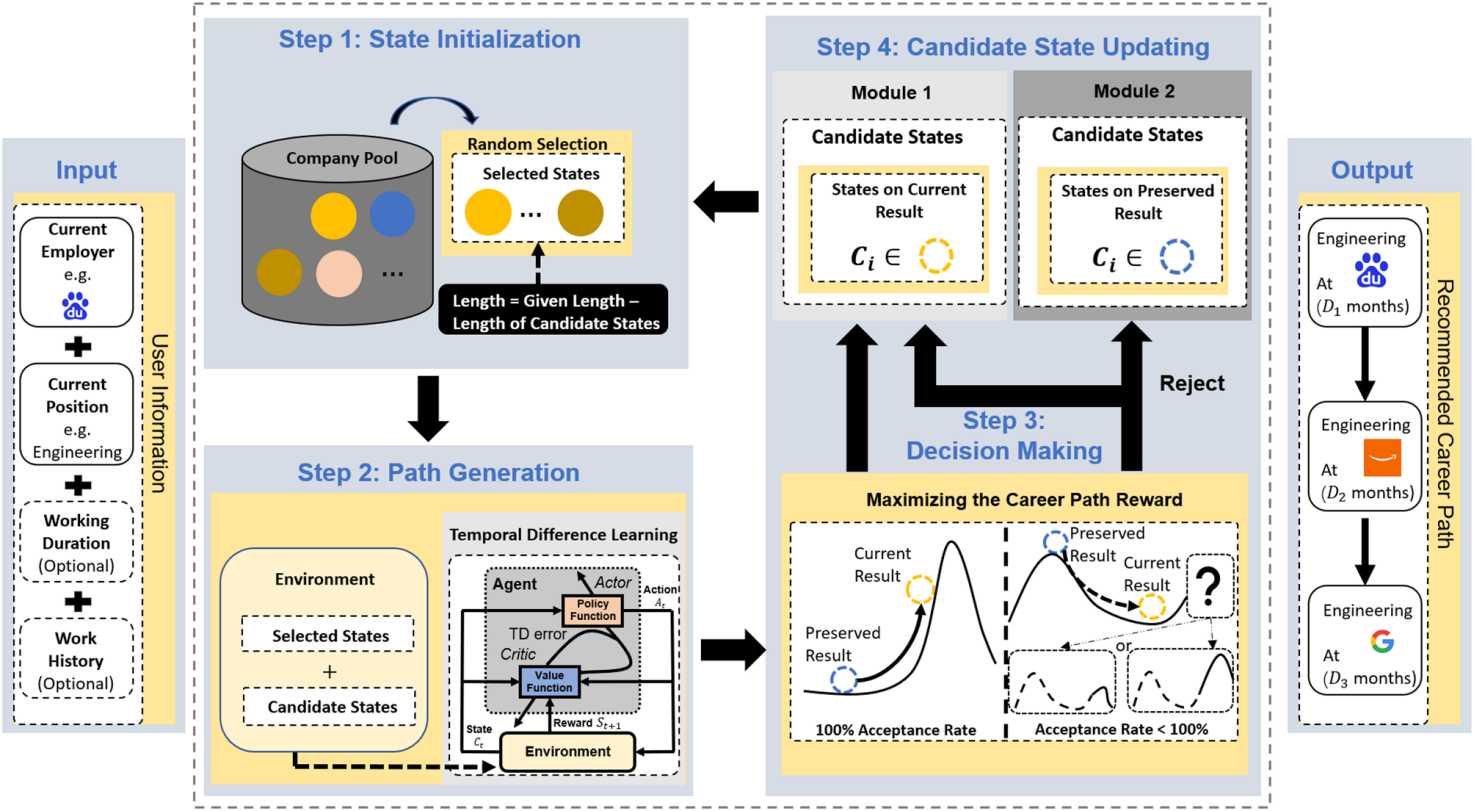
The SSRL framework. The inputs of SSRL contain user-provided information, including the current employer and position type as well as the optional working duration and work history information. Then, a four-step iteration handles the optimization process to provide personalized career guidance. Step 1 initializes the process and stochastically generates employer subsample based on the corresponding user states during the iteration. Step 2 handles RL environment construction and further performs the RL to explore optimal policy and generate the best career path based on the subsample. Note that the path evaluation function guiding the policy exploration jointly considers company and position features along with user preference and potential work experience gain in their career life. Step 3 determines whether to accept the current best path. To avoid being trapped in local optima, a cool-down strategy is proposed to allow accepting worse cases according to a probability following the Boltzmann distribution. Step 4 updates the candidate state accordingly and loops back to Step 1 for new subsampling. Once the terminating condition is met, SSRL will output the recommended career path.

The structure of our SSRL framework is demonstrated in Fig. 1. After taking the user inputs (e.g., the current employer, work history, etc.), the main component of SSRL consists of a four-step iteration. Step 1 of the SSRL handles the subsample generation from the original company pool $\mathcal{C}$ according to the candidate states. Step 2 involves the environment creating and module updating. Step 3 evaluates the path generated in Step 2 and determines whether the path is accepted. The update for the system’s candidate states is described in Step 4. If the model decides to accept the current result, then the candidate states will be updated according to the current result; otherwise, it remains the same as the preserved one. The optimal career sequence is finally located when reaching the predetermined number of iterations ($1\times 10^6$ in our paper).

Considering that the CSR problem is a long-term global optimization task, RL can be employed given its strength in offering long-term strategies. To do so, we view different companies $C_{i}$ as different states, and define a corresponding action $A_{i}$ that can be taken as the job hopping from company $C_{i}$ to $C_{i+1}$ after a duration $D_{i}$ with job $J_i$, and $S_{C_{i}}$ denotes the total accumulative reward for staying at company ${C_i}$ on $J_i$. Then, Eq. (1) can be rewritten as:

3 $$\begin{aligned} P_i = \left\{ S_{C_0}, C_{0}, A_{0}, S_{C_{1}}, C_{1}, A_{1}, S_{C_{2}},\ldots , C_{{n-1}}, A_{{n-1}}, S_{C_{n}} \right\} , \end{aligned}$$

where $C_{0}$ and $A_{0}$ denote the initial state and action.

Given the initial state $C_{0}$, to search for the optimal path $P^*$ generated by the optimal policy $\pi ^*$, we need to first determine the exploration strategy and the update rule. Since each state has a positive reward value, the greedy exploration strategies can hardly achieve the globally optimal selection, because they are usually dominated by the first selection of the current state in such cases. To address this issue, we encourage more explorations via an uniformly distributed random exploration strategy.

Regarding the update rule, for finite time and finite actions, the Q-learning^20^ is efficient in finding the optimal policy by updating the Q-table. As to indefinite time and finite actions, the dimension of the Q-table would blow up. To deal with the Q-table explosion, deep RL (DRL) was proposed to approximate the optimal action value and guide the action^16^. However, in our CSR problem, the career time is finite while the actions are indefinite, due to the huge and dynamic number of companies or organizations. Thus, limited by the space of the Q-table, the classical Q-learning method^20^ cannot be used. Meanwhile, the DRL will also fail due to parameter explosion, as the parameters of the deep neural network are linear to the size of the output layer, representing the number of available companies for job-hopping. That is, an indefinite number of companies would impede the utilization of DRL in which a fixed network structure is preferred.

Given that existing RL frameworks can hardly handle situations with indefinite actions in finite time, we propose a novel stochastic sampling method to shrink and stabilize the action space, and then apply a cool-down acceptance strategy to accelerate the exploration for global optima. The detailed pipeline of SSRL is shown in Algorithm 1.

In addition to long-term career planning, there are other potential practical implications of our method, where people need to make sequential decisions with an indefinite number of potential actions in a limited time. One example is the mobile sequential recommendation problem^21–23^, where a sequence of pick-up points are recommended for taxi drivers to follow to optimize their long-term benefit (e.g., maximizing the expected income, minimizing the expected driving time/distance). There are also other similar sequential problems in the literature, such as workflow optimization^24^, travel package recommendation^25^, training and skill development planning^5^, and so on. 
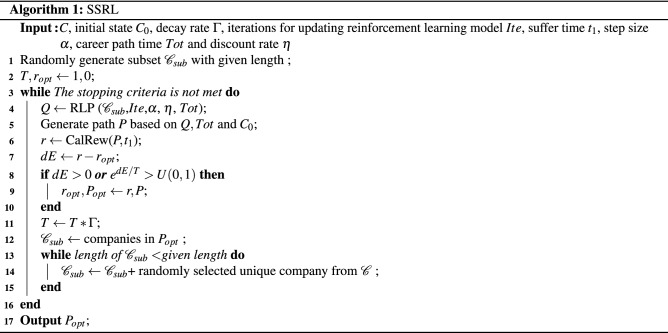


### Stochastic subsampling and cool-down accepting

Assuming that $\pi ^*_{\mathscr{C}}$ denotes the optimal policy on the company set ${\mathscr{C}}$, our strategy can be written as:

4 $$\begin{aligned} P^*=\underset{P_{\pi ^*_{\mathscr{C}_{sub_i}}}}{\mathrm{argmax}} \sum _{j \in P_{\pi ^*_{\mathscr{C}_{sub_i}}}} S_{j}, \end{aligned}$$

where $P_{\pi ^*_{\mathscr{C}_{sub_i}}}$ denotes the path generated by the policy ${\pi ^*_{\mathscr{C}_{sub_i}}}$, $\mathscr{C}_{sub_i} \subseteq \mathscr{C}$, and $S_j$ is a reward value for element *j*. The rationality and theoretical justification for this formulation have been provided in the Methods section.

Exhausting all possible optimal paths in Eq. (4) is expensive given the numerous company subsets. To accelerate the convergence, we propose to approach the global optima by gradually improving the local optima based on stochastically selected subsamples. Specifically, we explore the optimal path based on a randomly selected initial subset. Once the optima is achieved, we generate a new subset as the exploration pool. The process continues until the optimal path cannot be further improved with new subsets (see Proposition 2). To ensure that the model focuses on improving the current optima, we compose the subset ${\mathscr{C}_{sub}}$ by combining the companies on the current optimal path and other companies, which are randomly drawn (without replacement) from the full original company set ${\mathscr{C}}$.

However, due to the existence of several random components in our SSRL framework (e.g., duration estimator and position predictor), always rejecting the worse result may lead to trapped local optima^26–28^. To address this issue, we propose an acceptance determining step to select the optimal path via a cool-down strategy. Such process can be viewed as a Markov Chain Process (MCP). If a new path generated by the current optimal policy is better than the preserved one, we select the new path; otherwise, we choose between the new path and the preserved one, determined based on a probability following the Boltzmann Distribution. Formally, the decision parameter related to the acceptance probability $\omega$ can be shown as:

5 $$\begin{aligned} \omega = {\left\{ \begin{array}{ll} 1, &{} E_2-E_1>0\\ e^{\frac{E_2-E_1}{\Gamma ^{K}T}}, &{} E_2-E_1 \le 0 \end{array}\right. } \end{aligned}$$

where $E_{1}$ and $E_{2}$ are the accumulative rewards of the preserved optimal path and the new one, respectively; *T* is the temperature of the system; *K* denotes the decision making times, and $\Gamma$ is the decay rate which usually ranges from 0.9 to 0.99. If $\omega$ is larger than a random variable from zero to one, then the transition probability is equal to one, otherwise zero. We theoretically proved that the cool-down strategy can guarantee the convergence. During each iteration, SSRL updates the subset based on the selected optimal path. After a certain number of iterations, SSRL eventually locates an optimal career path for each individual (the convergence is proved in Proposition 3).

To locate the locally optimal policy ${\pi ^*_{\mathscr{C}_{sub_i}}}$, either Q-learning or deep reinforcement learning will work as the actions and time are finite. Since neural network is sensitive to its parameter setting and the structure^29–31^, we utilize Q-learning as our main method, and the following update rule is implemented:

6 $$\begin{aligned} Q(C_i,A) \leftarrow (1-\alpha )\cdot Q(C_i,A)+\alpha \left[ S_{C_{i+1}}+\eta \max _{a} Q(C_{i+1},a)\right] , \end{aligned}$$

where $\alpha$ denotes the step size ranging from 0 to 1; $\eta$ is the discount rate; $C_i$ represents the state; *A* is an action at current state leading to $C_{i+1}$, and $S_{C_{i+1}}$ is the reward at $C_{i+1}$. The detailed algorithm is shown in Algorithm 2. Note that, to show the generality of our SSRL framework, we also implement it with deep reinforcement learning as a benchmark. 
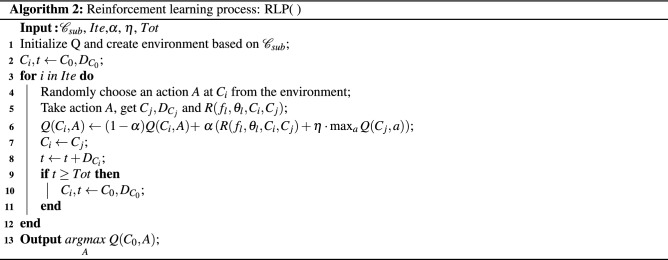


### Reward function for career path evaluation

The SSRL framework requires a carefully designed reward function to guide the exploration and evaluation of career paths. We formulate the reward by considering (1) the company rating, (2) the periodic extent of suffering, and (3) the staying probability. The company rating is determined by public features of companies (i.e., reputation, popularity, average staying duration and smooth transfer rate), and potential job position types (i.e., the extent of position matching). The periodic extent of suffering quantifies the negative effect of job-hopping on career paths. The staying probability measures how likely a person will move. Detailed settings and formulations of these features are discussed in the Methods section.

Mathematically, if we define *R* as a mapping function to the reward, the reward for staying in a company $C_i$ at a position $D_i$ with duration can be defined as:

7 $$\begin{aligned} S_{C_i}= R\left( \theta _l f_l,C_{i-1}, J_{i-1}, {C_i}, J_i, D_i\right) , \end{aligned}$$

where $f_l$ denotes the criteria of basic company features; $\theta _l$ denotes personal weights of each rating criterion; $C_{i-1}$ and $J_{i-1}$ represent the previous employer and corresponding job position, respectively. In our work, the periodic extent of suffering is determined by the similarity between $C_{i-1}$ and $C_i$; the staying probability is estimated based on company $C_i$ and the staying duration $D_i$. Algorithm 3 demonstrates the evaluating function for recommended path. 
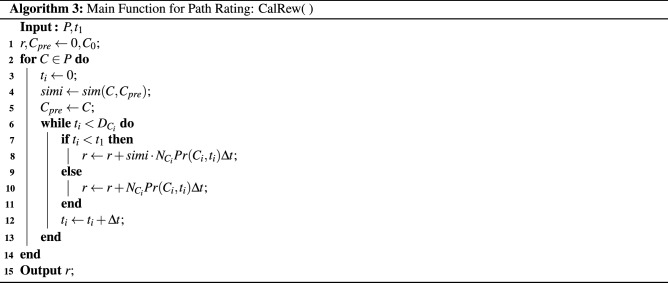


To increase the success rate of job hopping, instead of setting a single company at each state, our model can recommend a group of companies (company cluster) as a state. The reward of the cluster can be represented by the average reward of companies involved. Note that, with proper data resources, other factors associated with labor mobility may also be added to the reward function, such as industry and location^32^, position level^33^, income^9^, and many political and socioeconomic factors^34^.

## Results

We collected data from a famous online professional social platform, and our raw data contain over 40 million career records from more than 6 million randomly selected users and over 5000 companies. We removed observations with incomplete or missing features and some extreme cases. The cleaned dataset includes 6,495,600 users from 4281 companies and one company group. In our data there are over 500 companies that appear less than 5 times. To avoid biased measurements on their features, we consider them as the “other companies” group. Examples of the sequential structure of our data are provided in Table S.2, and important data statistics are reported in Table S.3 in the supplementary materials. The position type are classified into 26 categories following standard practice^12^. Our data involves the following limitations. First, our data do not include personal information (age, educational background, race, gender, etc.) and specific job information (e.g., job description, position level, salary, and specific work location. Second, given that the professional social platform users do not maintain their profiles in the same way, sampling bias may exist.

### The overall performance

We benchmark our model against five baselines, including three versions of “greedy” methods (i.e., JBMUL, IGM, and MGM) and two RL methods (TTD and PDQN). Note that our “greedy” baselines actually include the state-of-the-arts techniques for short-term recommendation tasks, while they are considered as greedy methods in a long-term view^35^.

To show the reliability of our method under different settings, We established four scenarios considering different user preferences. Scenario 1 is a general case where users do not have specific preference on company features. Scenario 2 is a personalized case where users consider company reputation as the most important aspect of potential new jobs. Scenario 3 is a personalized case where user preference changes over time. Scenario 4 is a personalized case where users have a clear plan for a specific period in their career life. Detailed settings of the four scenarios are provided in the Methods section.

**Figure 2:**
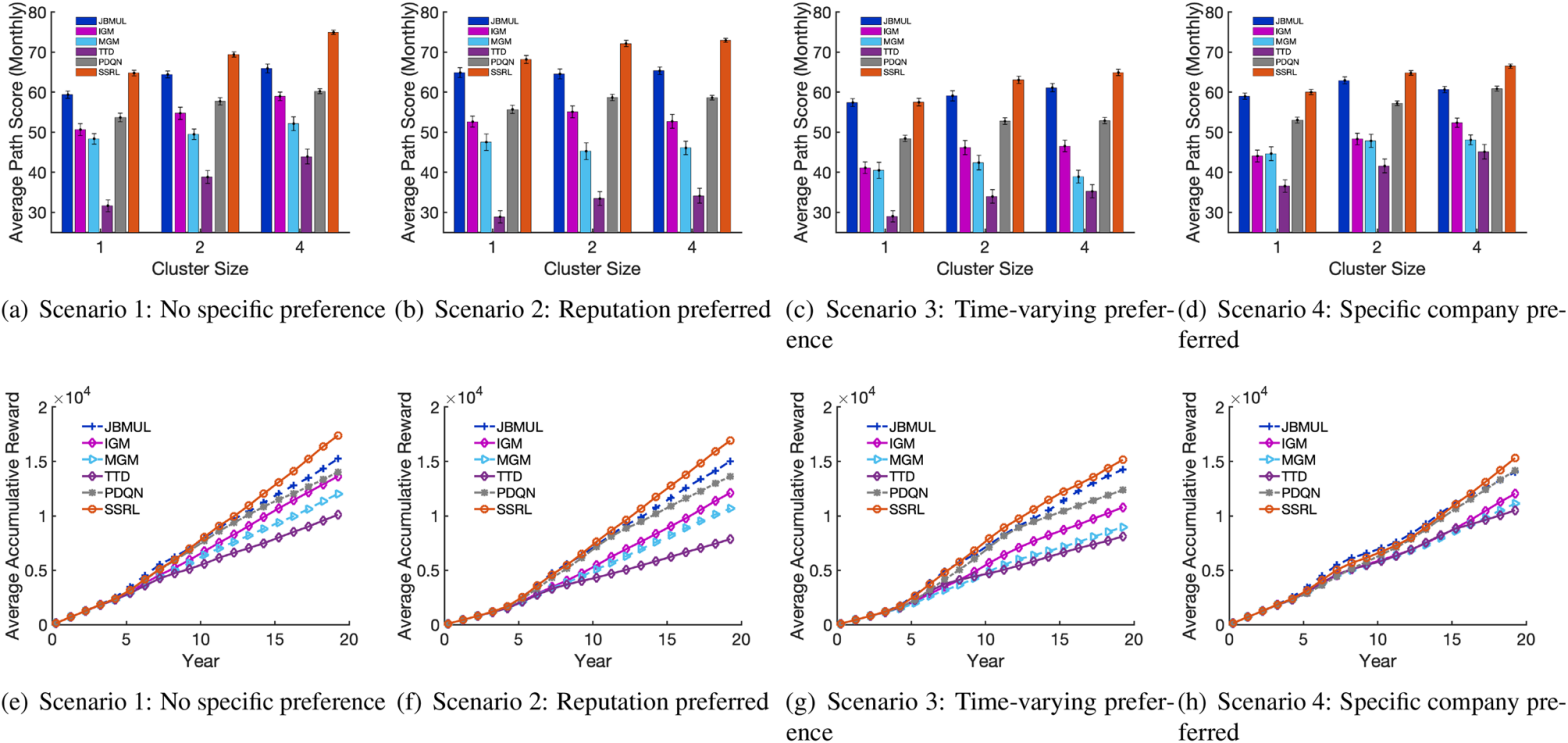
Average path score (per month) and accumulative reward. In this figure, (**a**)–(**d**) plot the average path scores (per month); (**e**)–(**h**) plot the accumulative rewards of recommended career paths over a 20-year career timeline ($\hbox {company cluster size} = 4$). Four experimental scenarios are tested. In Scenario 1, we consider a general case where people have no specific preference (i.e., the weight set of company-related features [*reputation*, *popularity*, *average staying duration*, *smooth transfer rate*] is set to [0.25, 0.25, 0.25, 0.25]. Scenario 2 represents a case where users may have a specific preference on one of the features, e.g., the reputation. The weight is set to [0.7, 0.1, 0.1, 0.1]. Scenario 3 mimics a dynamic preference in someone’s career life. We assume someone has a specific preference on the company reputation in the first 10 years (the same as Scenario 2), then the weight is updated to [0.1, 0.1, 0.7, 0.1], representing a preference change. Scenario 4 demonstrates an extreme case that someone has a preference on specific companies at a specific time. For example, someone has a clear plan to join Bank of Boston (or similar companies) at the early stage in her career (e.g., we lock our recommendations to the preferred companies at the third decision point [around the sixth career year]). For each case, we simulate the path with 30 random initial states, and the mean values are then plotted along with their standard errors [the error bars in (**a**)–(**d**)].

For the overall performance, Fig. 2(a)–(d) plot the average scores of recommended career paths under Scenarios 1–4. We ran the tests based on three different sizes of recommended company cluster (1, 2, and 4). All plotted values in Fig. 2 are based on 30 independent experiments. Under Scenario 1, the average career path score from the SSRL is 64.78 when setting the company cluster size to 1. Following the same company feature weight settings (equal weight), we compute the score of each career path in the real-world data. The average is only 41.96 (see supplementary Fig. S.1), representing the overall quality of career paths in real life without considering specific user preference information, and the SSRL shows a 54.3% improvement over it. From Scenario 2 to Scenario 4, the average path scores obtained from the SSRL are 68.20, 57.50, and 60.01, respectively. If four companies are recommended each time, the scores obtained from SSRL increase to 74.96, 72.97, 64.92, and 66.52, for Scenarios 1–4, respectively. Also, being consistently superior to all baselines, the results also suggest that our method can induce more advantage when allowing multiple companies to be recommended at the same time. In practice, more job recommendations would lead to more flexibility when facing the uncertainty of the future, hence a higher chance to secure a new job^36^. Moreover, the SSRL would produce the smallest standard error in all case settings, showing its consistent stability.

Regarding the baselines, the performance of the greedy methods (JBMUL, MGM, and IGM) is unstable under different scenarios. This is because they can be easily trapped in local optima while dominated by short-term benefits, given the non-convex situation. As expected, the classic reinforcement learning method TTD does not work well, due to its slow convergence rate when dealing with the CSR problem. PDQN is a deep RL jointly employed with our method. It leads to a better performance than TTD, indicating the flexibility of our method when applied to different model structures.

Furthermore, Fig. 2(e)–(h) plot the accumulative career path rewards on the 20-year career timeline with the company cluster size equal to 4. Based on our experimental settings, career path quality curves start diverging after 5 years staying at the initial company. As expected, although greedy methods (e.g., JBMUL) may have a better performance at the early stage, our SSRL can always achieve the best accumulative reward in the long run. In practice, greedy methods can be considered as similar strategy to human decisions, while machine processes more comprehensive data at each decision point. Our results indicate that the long-term advantage of the career planning method based on SSRL lies in the second half of the simulated career life.

### Case studies

We further investigate how our model guides and benefits people’s individual career life.

**Figure 3:**
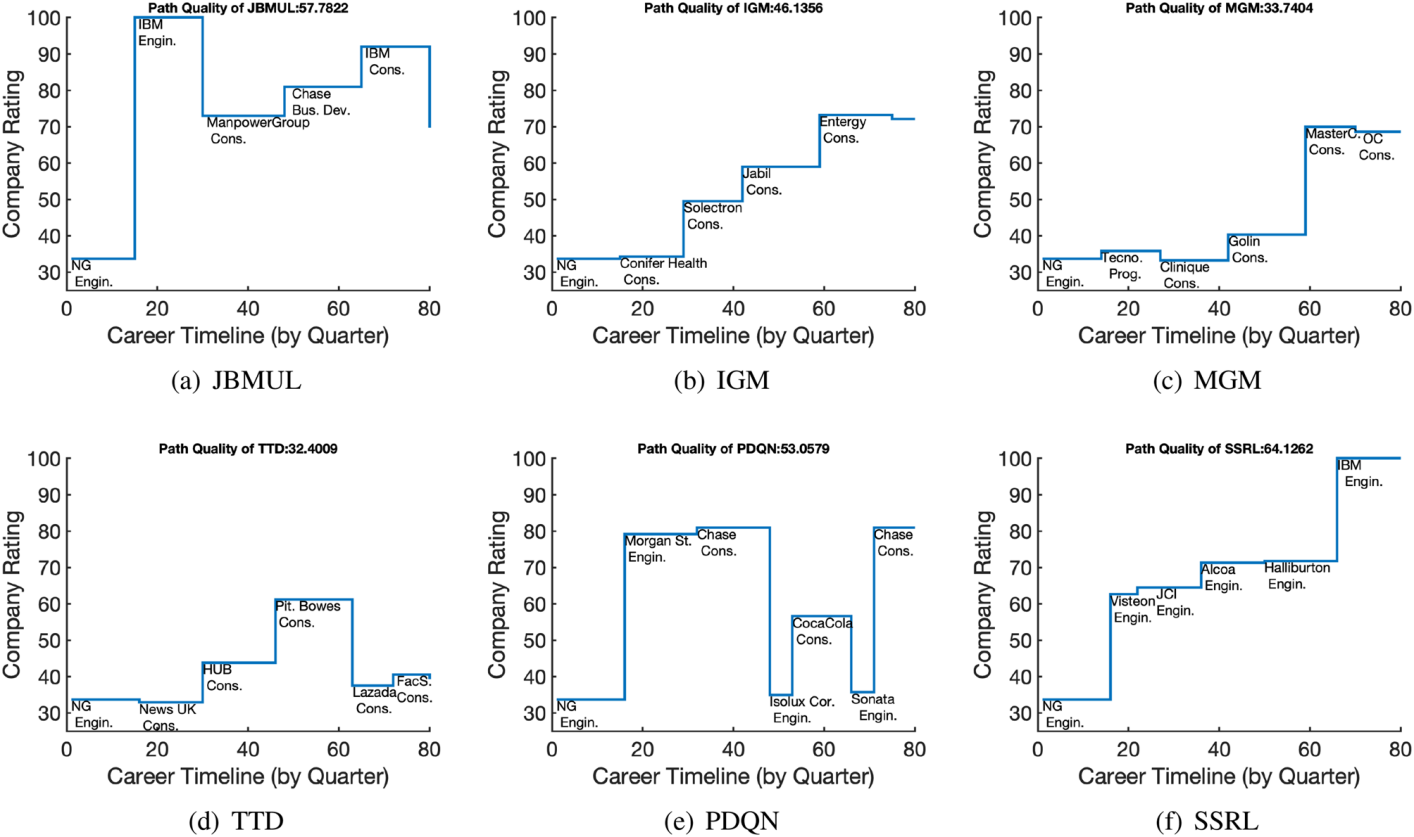
Career guidance. This figure illustrates career paths recommended by different methods, given the initial situation of an individual in the Navel Group as an engineer and with no specific user preference. We simulate 20-year career plans based on our method (SSRL) and five baseline methods. Compared to all baselines, SSRL shows a significant advantage in attaining the best-quality career path according to the pre-defined quality score. Also, SSRL recommended a gradually improved path, while all other baselines resulted in fluctuated quality of job mobility. If promotion and re-education information is not considered, SSRL tends to recommend the same-type of positions, while the companies may be from different industries.

*Case Study 1: Career Guidance.* To demonstrate detailed career planning guidance offered by our method and the baselines, Fig. 3 plots the recommended 20-year career paths, given an individual who starts her career at Navel group as an engineer. The simulation is done under Scenario 1 where people do not provide specific job/company preference. Three major findings regarding SSRL are summarized as follows. First, following the career path suggested by our method (SSRL), the user will achieve the best overall path score (64.13), which is about 11% better than the best baseline JBMUL’s 57.78. Second, SSRL’s recommendation suggests a gradually improved career life. During the 20-year career life, the user obtains improvement at every job change. Starting at the Navel group, the user finally joins IBM, which is undoubtedly a dream settlement in the engineering area. Facing the fast changing world, a gradually improved career path may benefit people by better overcoming the increasing number of life challenges given the uncertain future. Note that the measurement of career improvement can be subjective; hence, a good career path evaluation system should be customizable based on user preferences. Lastly, without considering promotion and re-education experience, our results show that SSRL suggests people stay in the same position type. This is reasonable because job type changes can easily cause lower work performance due to mismatched professional skills or experience^37^. On the other hand, the career path suggested by JBMUL involves several different position types. Still, it can be an excellent example of what most people would like to do in their career: getting into IBM as an engineer, then serving at consulting and business development positions in different companies, and eventually back to IBM doing consulting jobs. As discussed before, JBMUL is a short-term dominated method, while according to our setting, switching among position types negatively affects one’s career in the long-run view. Due to the data limitation, we did not consider promotion, further education information, and detailed job descriptions in our experiments. However, if given, our model can easily incorporate such information as additional factors in the reward function. It should be able to offer better career planning advice by considering reasonable mobility among different position types and constraints between skill categories^38^.

**Figure 4:**
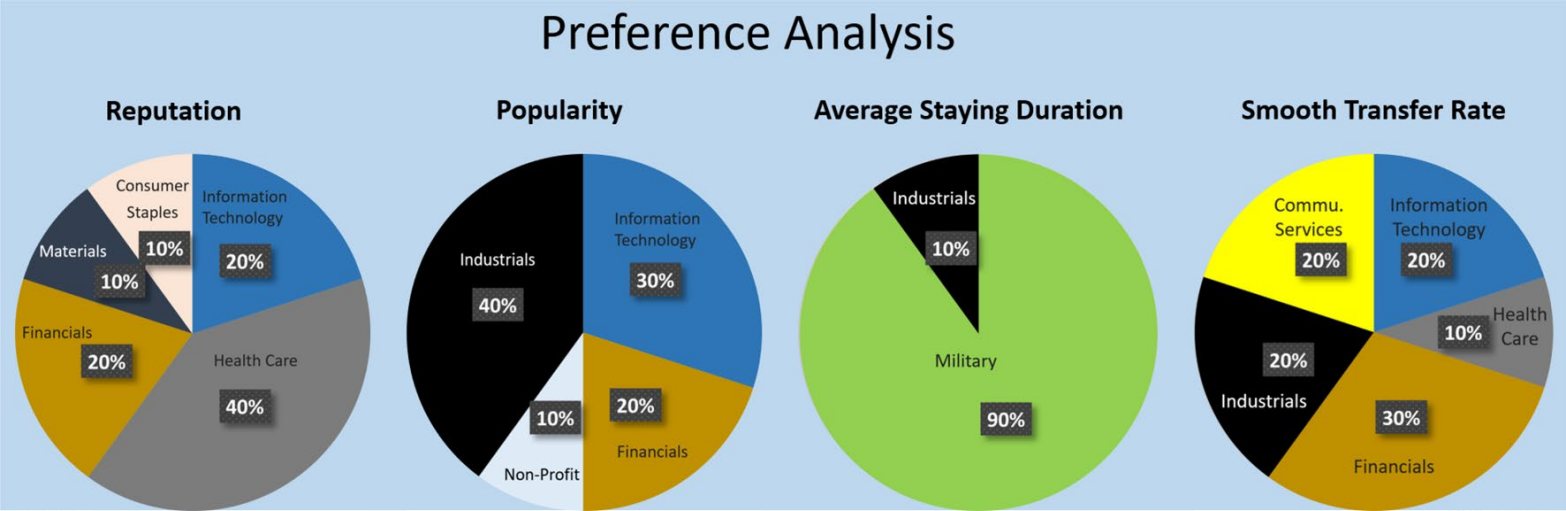
Business sectors of the top recommendations. This figure illustrates the portion of business sector of the top recommendations under different user preferences. The results are based on 200 career path simulations under different initial states. The weight for the preferred feature is set to be 0.7 while the rest being 0.1 each. We follow the Global Industry Classification Standard (GICS) to determine the business sectors.

*Case Study 2: Top Recommendations.* Now we demonstrate the top companies recommended by our model when people have different preferences on their new employers. Given the four company-related features, we set one of the user preference weights to 0.7 and 0.1 for the others. Then, we simulated 200 career paths with random initial states and report the top-10 most frequently recommended companies in Table 1. Also, following the Global Industry Classification Standard (GICS), the top recommendations are classified into different business sectors, and the percentage of sectors appeared are shown in Fig. 4. According to our results, 40% of the top recommendations for users who regard company reputation the most are in the health care sector (e.g., Pfizer). If popularity is the preferred feature, industrials (e.g., Accenture) occupy 40% of the top recommendations. In terms of the average staying duration, military jobs are on the top list (90% of the recommendation). Given that smooth transfer rate is preferred feature, among the top recommendations, financial firms are the most recommended sector (30%). We also provide supplementary results regarding the top-10 recommendations according to company feature-based scores under different user preferences (See supplementary results in Tables S.3 and S.4). Please note that our experiments are based on simplified scenarios where detailed job information (e.g., position levels, detailed job duties, qualification, etc.) is not considered. However, the model is flexible enough to take additional inputs and reflects which in the reward function. The exploration method SSRL is highly applicable to complex scenarios.

**Table 1:** Top-10 most frequent recommendations via SSRL.

	Preferred company/organization feature			
Rank	Reputation	Popularity	Average staying duration$^*$	Smooth transfer rate
1	Microsoft	IBM	HM Forces	Bank of America
2	IBM	Canon	Royal Air Force	IBM
3	Monsanto	Hewlett Packard	Indian Air Force	Pfizer
4	Pfizer	Accenture	U.S. Navy	Nortel
5	Walgreens Boots Alliance	Boys and Girls Clubs of America	Royal Navy	JPMorgan Chase
6	JPMorgan Chase	Bank of America	Royal Australian Navy	Northwest Airlines
7	Alcoa	Ernst and Young	Northwest Airlines	Lockheed Martin
8	General Motors	Microsoft	Royal Australian Air Force	Level 3 Comm.
9	UnitedHealth	JPMorgan Chase	U.S. Air Force	Sun
10	BMS	PwC	British Army	UnitedHealth

This table reports the most frequently recommended companies or organizations via our SSRL framework, based on different user preferences on the four company-specific features in our evaluation model. *Given that the “average staying duration” is the preferred feature, the top-10 recommendations obtained include many military positions. Military is not a sector defined by GICS. Thus, we consider it as a spacial class. If removing military positions, the top-10 recommendations are: *Northwest Airlines, Electronic Data Systems, Various Inc., Delta Air Lines, Kodak, Texas Instruments, Carlson Marketing, Bell Laboratories, Ford Motor Credit Company, CGG.*

## Discussion

Does a person’s first job matter? To answer this question, we generate career path recommendation via the newly proposed stochastic subsampling reinforcement learning (SSRL) framework with different initial companies and position types. Four initial companies (two with average ratings and two with high ratings) and four popular job types are selected to form the initial states, resulting in 16 combinations in total. We do not consider specific user preference in these simulations. Note that the company ratings in our work are computed based on simplified initial settings, hence they do not reflect real company quality. Based on our model, a company may present different ratings, given diverse user preferences and the degree of job-person fit at different stages in one’s career life. In Table 2, Panel A summarizes the average path scores and standard errors (in parentheses) based on 30 independent simulations for each case setting. It turns out that initial companies with similar ratings (e.g., Barcelo and ACC; AstraZeneca and Fedex Office) would lead to closely scored career paths. Also, our results reveal a trend that a higher-rating initial company would usually lead to a higher-score career path; whereas, given the large gap between the initial company ratings, we find reduced differences between the scores of corresponding career paths suggested by the SSRL. This indicates the strength of our method in seeking the optimal path for users given varying initial career states. Furthermore, in Panel B of Table 2, we investigate the portion of “Good Paths” in the simulated career paths. Given the average path score in the real-world (human-decision) data (41.96) and its standard deviation (12.33), a “good career path” is defined as one with path score larger than 66.62, which is the top 2.2% based on the Gaussian distribution. As expected, better initial states in people’s career are more likely to end up with “successful” career paths, according to the good path percentages obtained. This may lead to another interesting research direction that we intend to address in follow-up work. That is, how training and education can be optimized to lead to a jump start in one’s career, based on personal characteristics and given limited social and private resources.

**Table 2:** Career path planning based on different initial companies and positions.

	Initial position type			
Initial company [rating]	Legal	Sales	Engineering	Support
Panel A: Average path score				
Barceló [34.00]	60.34 (0.65)	59.48 (0.50)	60.30 (0.52)	59.33 (0.59)
ACC$^*$ [34.08]	59.92 (0.51)	60.09 (0.52)	60.60 (0.54)	59.31 (0.50)
AstraZeneca [62.35]	67.52 (0.52)	66.51 (0.51)	68.62 (0.45)	67.54 (0.50)
FedEx Office [62.44]	68.77 (0.33)	67.28 (0.63)	68.05 (0.46)	67.29 (0.58)
Panel B: Good path percentage				
Barceló [34.00]	0.03	0.00	0.00	0.03
ACC$^*$ [34.08]	0.00	0.00	0.00	0.00
AstraZeneca [62.35]	0.57	0.60	0.73	0.67
FedEx Office [62.44]	0.87	0.60	0.70	0.60

This table summarizes the average path scores and good path percentage based on experiments with different initial companies and position types. We selected four companies (two average-rating companies and two high-rating companies) and four position types, leading to 16 combinations for the investigation. For each combination, 30 independent experiments were conducted. Panel A reports the average path score of the recommended career paths, along with corresponding standard errors (in parentheses). Panel B reports the “good path” percentages based on the same experiments. We define a good career path as one with a score greater than 66.62. *ACC: American Campus Communities Inc.

Another critical matter in AI-based decision systems with increasing awareness and research is the ethical issue. How can these systems consider users’ feelings when making decisions for them? Existing job mobility data usually do not contain features to measure people’s feeling during work. Being trained on historical data, algorithmic decision-making tools may replicate bias in the past^39,40^. As suggested in reference^41^, transparency and de-biasing techniques are essential to address bias-related ethical issues in AI-based decision support systems. Given the difficulty in quantifying feelings, which can be affected by many factors (e.g., personality, growing environment, personal experience, etc.), we believe it is still too early to introduce a pure AI-based system to make life decisions for human. The AI methodology we have proposed in this work only seeks to help people better understand themselves by demonstrating a potentially optimal career path in the long-run view. We strive to make the system flexible in considering user preference by pre-defined parameters, such as the weight of company rating factors. Moreover, comparing with deep learning, RL-based systems have the advantage in transparency as their training process can be backtracked. We believe the existence of such AI applications will benefit human society by illustrating people’s long-term potential, hence aiding them make important life decisions more rationally and deliberately.

Moreover, in addition to the de-biasing component, another research direction of AI-based systems is to offer better support to those who can be affected more (e.g., women and caregivers^42,43^) during a pandemic or other natural disasters. Good AI systems should be able to detect changes in circumstance and offer adaptive decision supports^44^.

## Methods

This section discusses the reward function in SSRL and provides theoretical details of the search algorithm for the optimal career path. We summarize important notations and their definitions in the table shown in the supplementary materials.

### Reward function

Now, we discuss the formulation of our reward function with three major components including company rating, periodic extent of suffering, and staying probability.

*Company Rating.* The company rating is formulated as a linear combination of *company feature-based score* and *position feature-based score*.

First, we introduce the company feature-based score, which is used to evaluate the overall “quality” of a company. The following four factors are considered.

1. *Reputation*: The reputation of a company is considered a representative factor of its business value and social impression, hence will significantly affect the job stability of employees. By investigating a sample containing 593 large publicly traded companies from 32 countries, Soleimani et al.^45^ found a positive impact of company reputation on its stock performance and the employee salary. Similar findings can also be found in^46^. It has been found that people are more willing to work in companies with high reputation^47^. In this paper, we quantify three levels of company reputation. For the first (highest) level, Fortune-500 companies are rated with the highest reputation, and their reputation score is set to 1. Non Fortune-500 companies with more than 10,000 employees are placed to the second level, with a reputation score $\frac{2}{3}$. For the rest of the companies, we assign $\frac{1}{3}$ as the reputation score. We also conducted experiments to evaluate the stability of the overall performance based on linear reputation. Related results are included in the supplementary materials (Fig. S.2).
2. *Popularity*: Popularity represents the overall social impact of a company. Based on experiments on an artificial market, Salganik et al.^48^ suggested that popularity is positively related to the quality of a company, and people are more willing to work in popular companies. Company popularity can be easily quantified based on talent move record. The frequency of talent incoming transfer indicates the popularity of a company. In our work, we normalize the total number of incoming transferring records to values in [0, 1], by dividing each by the maximum of the records.
3. *Average Staying Duration*: Employee stability is essential to business success^49^, and the average staying duration of a company also represents the overall job satisfaction of its employees^50^. We compute the average staying duration of employees for each company and then normalize them to [0, 1] based on the maximum value.
4. *Smooth Transfer Rate*: The smooth transfer rate measures how likely a job-hopping can be made. Given the dynamic market, a smooth job hopping indicates less risk hence is preferred by most job seekers^6^. Considering the labor market shifts toward information and knowledge based work, talented workers are the intangible asset to a company^37^. From the perspective of employers, to keep the competitive advantage, companies should accept suitable employees as soon as possible. To measure the smooth transfer rate, we have the following settings. For those companies with more than 100 transfer records, we calculate it as the ratio of the number of transfer records without waiting time (no time gap between the old and the new jobs) over the total amount of job transfers. For companies with less than 100 transfer records, which occupy only 3% of the full sample, we introduce a penalty term (0.8) to weaken the smooth transfer rate, as the number may be overestimated due to a small sample size.

Importantly, considering the fast changing labor market, company ratings may vary over time^5,6^. The requirement from users is also changing over time. Assuming that ${t'}$ denotes the time people start to work at the company $C_i$, based on the above four features indicated by $f_l$ where *l* is the feature index from 1 to 4, the *company feature-based score* of company $C_i$ is defined as:

8 $$\begin{aligned} N_{C_{i_{CF}}}^{t'}=100\cdot \frac{\sum _{l=1}^4 \theta _l^{t'} f_{l,C_i}^{t'}-\min _{j \in \mathscr{C}} \sum _{l=1}^4 \theta _l^{t'} f_{l,j}^{t'}}{\max _{j \in \mathscr{C}} \sum _{l=1}^4 \theta _l^{t'} f_{l,j}^{t'}-\min _{j \in \mathscr{C}} \sum _{l=1}^4 \theta _l^{t'} f_{l,j}^{t'}}, \end{aligned}$$

where $\mathscr{C}$ denotes the company list and $\theta _l$ denotes the weight of features. Users can express his/her preference to each company feature-based by specifying $\theta _l$. Note that $N_{C_{i_{CF}}}^{t'}$ ranges from 0 to 100 based on the formulation.

On the other hand, we estimate the position feature-based score as follows. The job position contributes to one’s company rating in terms of the person-job fit, which is found positively correlated to job satisfaction^51^. From the perspective of employers, job seekers are also encouraged to be matched to a background-fitting position^37^. The position for the next company is related to the employee’s current experience. In this paper, the new job position is predicted by a job predictor based on the records in our dataset. The job predictor is determined by counting all the position transfer records for the current position and normalizing the top three most frequently selected positions. If the job position is the same as before, then people will get full credit for this position. If the position type is changed, we further evaluate the new work environment (i.e., whether employees get enough team support during the learning/training period).

As expert is instrumental when people face new difficulties in the team^52^ and the support from team is vital for overcoming the difficulty^53^, we assume that if people can get enough support from expert team members, the new job is desirable even if it is a new position type. The number of experts in the given position should be positively related to the number of the positions in the company. Thus, to evaluate if people can obtain enough support from the team, we measure if there are sufficient number of co-workers at the same job position in the company. If the job is among the top-3 major position types in the company, we assume that people are more likely to receive sufficient support; otherwise, insufficient support is assumed. To differentiate the above cases quantitatively, we have the following settings. Assuming that the previous position is $J_{i-1}$ and the current position is $J_i$, the *position feature-based score* for company $C_i$ can be defined as follows:

9 $$\begin{aligned} N_{C_{i_{PF}}}= {\left\{ \begin{array}{ll} 100, &{} J_i=J_{i-1}\\ 80, &{} J_i \ne J_{i-1}; J_i \text { is among the top-3 position types in } C_i \\ 60, &{} \text {otherwise} \end{array}\right. } \end{aligned}$$

Please note that this is a simplified formulation we use to assess the overall person-job fit. More complicated formulation can be adopted in real cases.

Given company feature-based score $N_{C_{i_{CF}}}^{t'}$ and position feature-based score $N_{C_{i_{PF}}}$, we define the company rating of $C_i$ as their linear combination:

10 $$\begin{aligned} N_{C_i}=\beta _1\cdot \left( s_1\cdot N_{C_{i_{CF}}}^{t'}+s_2\cdot N_{C_{i_{PF}}}\right) , \end{aligned}$$

where $s_1$ and $s_2$ are the weights of the two feature scores with $s_1 + s_2=1$. Here $\beta _1$ is used to describe a negative effect of downtrend career moves on the company rate. People usually seek opportunities to work in better companies with improved work environment and potentials^54^. It has also been found that people would have a decreased job performance with their new employers if the work environment did not improve^37^. Thus, we have the following settings: $\beta _1<1$ if $N_{C_i} \le N_{C_{i-1}}$; $\beta _1 = 1$ if $N_{C_i} > N_{C_{i-1}}$.

*Periodic Extent of Suffering.* Human capital transferring is not easy, and sometimes will lead to a job performance reduction^37^. We refer to this as the suffering period after job hopping. According to a survey presented by Morse and Weiss^55^, people are more willing to work in the same type of companies as their current one when seeking the next job. Evidence was also found that firm-specific skills plays an important role in employees’ performance^37^. It is common that companies in the same business sector have similar positions (e.g., *JP Morgan Chase* and *Bank of America*). Thus, we estimate the extent of suffering by the similarity of the current and potentially new companies. A higher level of similarity is supposed to result in less suffering in the new company.

Let *j* be the order index of a given position type list, the percentage of the $j^{th}$ position type in company $C_i$ is computed as:

11 $$\begin{aligned} Per(C_i,j)=\frac{\big |\{PT({C_i,k})= j|_{k=1,2,\ldots ,M_{C_i}}\}\big |}{M_{C_i}}, \end{aligned}$$

where $PT({C_i,k})$ is the position type of the $k^{th}$ position in company $C_i$; $M_{C_i}$ is the total number of position records in company $C_i$. Then, we compute the cosine similarity between companies $C_i$ and $C_{i-1}$ as follows:

12 $$\begin{aligned} sim(C_i,C_{i-1})=\frac{\sum _{j=1}^{n_p} \left[ Per\left( C_i,j\right) Per\left( C_{i-1},j\right) \right] }{\sqrt{\sum _{j=1}^{n_p} [Per\left( C_i,j\right) ]^2}\sqrt{\sum _{j=1}^{n_p} [Per\left( C_{i-1},j\right) ]^2}}, \end{aligned}$$

where the total number of position types $n_p = 26$ in our data.

*Estimator for Staying Probability and Duration.* The staying probability not only helps estimate the duration, but also serves as an important component in our reward function. The following equation offers a simple way to estimate the staying probability for a given company:

13 $$\begin{aligned} Pr(t)=1-\frac{d(t)}{n(t)}, \end{aligned}$$

where *d*(*t*) denotes the number of people left the company before time *t*; and *n*(*t*) represents the total number of employees in the company and $n(t) \ge d(t)$.

However, such estimation may be inaccurate due to data noise and incomplete records from existing employees. For a current employee in a company, we can only define his/her future situation as unknown or uncertain. To obtain a more reliable staying duration, we estimate the staying probability for such samples via survival analysis. Our problem can be considered as a right-censoring condition, given that only the starting time of each job is known. Thus, we apply the Kaplan–Meier (KM) estimator from survival analysis. Specifically, we define staying at a company as “survive”, and leaving the company as “die”. Let *d*(*x*) denotes the number of “survivers” from $[x, x+\Delta x)$ and *n*(*x*) denotes the number of individuals at risk just prior to a given time *x*; i.e., the number of individuals in the sample who neither left nor were censored prior to time *x*. Then, the KM estimator for the staying probability can be written as:

14 $$\begin{aligned} Pr(t)=KM(t)= \prod _{x \le t} \left( 1-\frac{d(x)}{n(x)}\right) , \end{aligned}$$

where $x \le t$ represents all grid points such that $x+\Delta x \le t$. See technical details of KM estimator in reference^56^.

The staying probability can be used to estimate the staying duration of employees. It has been found in the literature that people tend to stay in similar type of companies in their career life^57^. As the estimated staying probability represents a mainstream pattern, we have the following design to differentiate mainstream followers from others. Given a current company, we define the mainstream selection for the next employer as the top-100 most selected companies in the data. Also, if a person chooses her new job from the mainstream list, the remaining companies on the list continue as the mainstream choices for the next job-hopping. Suppose a person is working at her $i^{th}$ company, and $\Psi (C_i)$ is the set of top-100 job-hopping selections for people who worked at company $C_i$. The mainstream choices for the $(i+1)^{th}$ job-hopping is defined as $\Psi (C_i) \cup \Psi (C_{i-1}) \cup \Psi (C_{i-2}) \cdots \cup \Psi (C_0)$. Importantly, this design enables us to partially simulate the age and learning experience effects, which have been found to be important factors of career mobility^58^. In the short-term view, we assume that there will be more useful experience gain for the mainstream followers. Off the mainstream may indicate lower survival rate due to irrelevant experience. On the other hand, in the long-term view, if people try different types of companies/positions and received “panelties” in their early career stage, there will be a broader range of “mainstream” companies that they can survive in the later career stages.

Therefore, one’s staying duration at company $C_i$ can be estimated according to $\beta _2 Pr(C_i, t)$, where $\beta _2$ is a penalty term, which is set to 1 if $C_i \in \Psi (C_i) \cup \Psi (C_{i-1}) \cup \Psi (C_{i-2}) \cdots \cup \Psi (C_0)$, or 0.8 otherwise. The staying duration can be easily determined when the model concludes the “leaving state” for a current company $C_i$ based on $\beta _2 Pr(C_i, t)$ at time *t*.

*Reward Formulation: The Final Form.* To find the optimal path $P^*$, we need to define a proper reward function *R* to guide the exploration and determine the optimal path. We define the reward as the accumulative score considering the company rating, the periodic extent of suffering, and the staying probability. Thus, the total reward for staying at company $C_i$ is defined as:

15 $$\begin{aligned} S_{C_{i}}= & {} \int _{0}^{t_1} N_{C_i}\cdot Pr(C_i, t) \left( sim(C_i,C_{i-1})-1\right) dt\nonumber \\&+\int _{0}^{D_{C_i}} N_{C_i}\cdot Pr(C_i, t) dt, \end{aligned}$$

where $t_1$ is the suffering period; $N_{C_i}$ is the company rating; $D_{C_i}$ means the duration at company $C_i$; $Pr(C_i,t)$ denotes the staying probability for company at time *t*; and $sim(C_i,C_{i-1})$ is the similarity between two companies.

Equation (15) evaluates the reward for staying at company $C_i$. The first component $\int _{0}^{t_1} N_{C_i}\cdot Pr(C_i, t) \left( sim(C_i,C_{i-1})-1\right) dt$ measures the weakening effect during the suffering period at a new company. The second component $\int _{0}^{D_{C_i}} N_{C_i}\cdot Pr(C_i, t) dt$ estimates the accumulative reward with decayed staying probability. To ease the computing, we divide the total time into small intervals $\Delta t$, and then Eq. (15) can be reformulated as:

16 $$\begin{aligned} S_{C_{i}}&= R\left( \theta _l f_l,C_{i-1}, J_{i-1}, {C_i}, J_i, D_i\right)\\ &= N_{C_i}\left( \left( sim(C_i, C_{i-1})-1\right) \sum _{k=1}^{t_1/\Delta t} Pr(C_i,k \Delta t) +\sum _{k=1}^{D_{C_i}/\Delta t} Pr(C_i,k \Delta t)\right) \Delta t \end{aligned}$$

### Theoretical analysis

In the following, we provide further analysis on our SSRL framework, focusing on the properties of the proposed CSR problem, our exploration strategy, the speedup of convergence, and their theoretical supports.

*Fundamental Property.* Given the starting state $C_{0}$, assuming that we have found the optimal path $P^{*}$ generated by the optimal policy $\pi ^*$, then we should have the following property.

#### **Property 1**

(Upper Bound of Policies) *Given finite career time, if* $P^*$ *is the optimal career path starting with* $C_{0}$, *we should have* $\sum _{j \in P_i} S_j \le \sum _{j \in P^*} S_j$, *for any path* $P_i$ *with the same initial state and career time length*.

All proofs can be found in the supplementary information. According to our problem setting, the unpredictable size of the company set $\mathscr{C}$ may result in huge computing challenges, while our solution is to achieve the global optimum with fixed-sized subsets $\mathscr{C}_{sub}$, leading to the following lemma as a special case of Property 1.

#### **Lemma 1**

(Upper Bound of Local Policy) *Given an initial company* $C_0$, *for any optimal paths generated by* $\pi ^*_{\mathscr{C}_{sub}}$ *the optimal policy on* $\mathscr{C}_{sub} \subseteq \mathscr{C}$, *their accumulative rewards cannot exceed that of* $P^*$ *generated by the optimal policy* $\pi ^*_{\mathscr{C}}$.

Lemma 1 demonstrates a challenging optimization task in our work, which is to achieve the global optima based on local exploration. That is, our SSRL needs to explore the global optima based on local policy $\pi ^*_{\mathscr{C}_{sub}}$. The following proposition defines the sufficient and necessary condition of this task.

#### **Proposition 1**

(Boundary Condition) *Suppose that we define* “*target companies*” *as those appear on the globally optimal career path*. *The global optima can be achieved by a local policy* $\pi ^*_{\mathscr{C}_{sub}}$, *if and only if the target companies are included in the corresponding company subset* $\mathscr{C}_{sub}$.

Proposition 1 indicates that it is possible to locate the global optimum by exploring the best locally optimal policy, instead of exploring the whole company pool. Our exploration method is designed to handle global optimization task by stochastically exploring local optima. Please note that the target companies cannot be discovered directly, while Proposition 1 guarantees that the target companies are included in the final company subset if the global optima is achieved.

*Exploration strategy.* Assuming that there are *m* companies in a company subset, the number of candidate subsets equals to $\frac{N!}{m!(N-m)!}$. Given a large total number of companies *N*, it is still expensive to find the best path generated by the local policy, if we have to explore all candidate subsets. As we aims to find the best locally optimal path, we have the following proposition.

#### **Proposition 2**

(Transformation Condition) *With a large number of iterations, if the quality of the optimal path cannot be further improved based on randomly generated subsets, then the current optimal path is the global optima*.

According to Proposition 2, as long as the policy is continuously optimized in a stochastic process, we will obtain the best local optima eventually. Thus, we need to make sure the exploration process will converge with a time limit. As we introduce several random events in our model (e.g., duration estimator, position predictor), the result for current optimal policy might be worse than the preserved one due to the uncertainty in the path generation. To accelerate the convergence, we develop a cool-down strategy based on the Boltzmann distribution [see Eq. (5)].

Cool-down strategies have been developed with simulated annealing techniques and are found efficient to handle sequential recommendation tasks^22,28^. In this paper, the cool-down strategy is developed under a more complicated RL-based framework to speed up the career path exploration. Theoretically, the convergence is guaranteed, referring to our Proposition 3 as follows.

#### **Proposition 3**

(Convergence analysis) *The cool-down strategy guarantees the convergence under the uncertain scenario in career path recommendation*.

### Baseline methods

Five baselines were implemented in this work, including JBMUL, IGM, MGM, TTD, and PDQN. We summarize their advantages and disadvantages in Table 3.

- *JBMUL.* We follow the idea of reference^59^ to locate the best result at each time step. For selecting the next state, we calculate the accumulative reward of each company based on the current state. Then the model selects the maximum accumulative reward as the next state and continue.
- *IGM*. This method aims to find the result which is better than the previous state. For the current state, we calculate the accumulative reward of each company. Comparing the result with previous state’s reward, as long as it is better than or equal to the previous one, we will place the company on the current state and continue to the next state.
- *MGM*. This method is designed for finding the optimal solution based on the average accumulative reward of accepted companies. For the current state, as long as the calculated reward is better than or equal to the average reward of accepted companies, we will accept this allocation and continue to the next state.
- *TTD*. As mentioned in reference^60^, TTD is a traditional off-policy temporal difference learning. After a fixed number of iterations, TTD will generate the policy based on the initial state.
- *PDQN*. This baseline evaluates the implementation of our exploration strategy in deep RL. We regularize the action based on our exploration strategy (i.e., subset generation) and update the policy by the deep RL. We deploy a simple neural network with one hidden layer, the input and output size is set to 20, and the size of the hidden layer is 40.

**Table 3:** Comparison of benchmark methods.

Method	Description	Advantage	Disadvantage
JBMUL	Traditional greedy method	Efficient for convex situation	Trap into local optimal easily in non-convex situation
IGM	Modified greedy method	Available to leave local optimal	Convergence rate is slow
MGM	Modified greedy method	Available to leave local optimal	Performance is unstable
TTD	Traditional RL method based on Q-table	Efficient for long-term sequence decision	Limited by the size of actions and states
PDQN	Advanced RL framework based on neural network	Efficient for long-term sequence decision with infinite time.	Limited by the size of actions

### Experimental settings

We set the time length of career path to 20 years and the precision of time interval $\Delta t$ is a quarter, the suffer time is set to 1 year. The default setting for the optional information in the input (e.g. working duration, work history) is none. For RL method, the discount rate $\eta = 0.9$, and the step size is set to 0.01. The total number of iterations is set to 1,000,000 steps; and if the total work time is more than 20 years, we will restart at the initial state. For SSRL and PDQN, we set the initial temperature *T* to 1, and the decay rate $\Gamma = 0.99$. The fixed length of the subset $\mathcal{C}_{sub}$ is set to 20, and we compare the result every 100 iterations. For each method, we simulate the career path on different numbers of processors and average the path score under the same grading criteria.

The major setting of the four scenarios in our experiments are related to the weight of the company-related features, including reputation, popularity, duration, position change rate and smooth transfer rate.

- *Scenario 1.* General case (no specific user preference). This case shows the general case of our recommendation in which the user has no preference for the company features. The weights for all the features are 0.25.
- *Scenario 2.* Personalized case (reputation preferred). This case indicates the path for those users who care more about the company reputation. The weight for the reputation is 0.6 and others are 0.1.
- *Scenario 3.* Personalized case (time-varying preference). This case reflects the dynamical requirements from user at different career stage. The weights for the first half of career path are [0.1, 0.1, 0.1, 0.7] while the rest are [0.1, 0.7, 0.1, 0.1].
- *Scenario 4.* Personalized case (specific company preferred). This case simulates the situation that user has a rough plan for his/her career. We set the plan as being transferred to the Bank of Boston at the third career stage for 4 years.

### Experimental environment

All experiments were conducted on a high-performance computer (HPC) cluster with 164 compute nodes, each with two Intel Xeon E5-2683v3 20-core processors and 128 GB DDR4 Memory.

## Supplementary Information

Supplementary Information.

## Data Availability

Data of this paper are available via https://1drv.ms/u/s!Ajf4p4o3pDQGdPr6cPX-XeCKxRE?e=RfU6zt.

## Code availability

Codes of this paper are available in CodeOcean https://codeocean.com/capsule/9b8bcace-e6cb-4842-aaa9-c4e1443a8cbd/.
