# Supplementary material for: Intelligent career planning via stochastic subsampling reinforcement learning
Source: Sci Rep. 2022 May 18;12:8332. doi: 10.1038/s41598-022-11872-8 (PMC9117248; doi:10.1038/s41598-022-11872-8)
Supplement: Supplementary file 1 — Supplementary Information. [file 41598_2022_11872_MOESM1_ESM.pdf]

Intelligent Career Planning Via Stochastic Subsampling Reinforcement Learning (Supplementary Materials)

Pengzhan Guo[1], **Keli Xiao**[2,*], **Zeyang Ye**[3], **Hengshu Zhu**[4,*], **and Wei Zhu**[2,*]

[1]Duke Kunshan University, Kunshan, Jiangsu, China
[2]Stony Brook University, Stony Brook, NY, USA
[3]Samsung Research America, Mountain View, CA, USA
[4]Baidu Talent Intelligence Center, Beijing, China
[*]Corresponding authors (keli.xiao@stonybrook.edu, zhuhengshu@gmail.com, wei.zhu@stonybrook.edu)

List of Supplementary Materials

Supplementary Note 1: Additional Related Work

Supplementary Note 2: Important Notations and Definitions

Supplementary Note 3: Theoretical Proofs

Supplementary Note 4: Data Format

Supplementary Note 5: Important Data Statistics

Supplementary Result 1: Distribution of Average Staying Duration and Path Quality

Supplementary Result 2: Top-10 High-Score Recommendations under Different User Preferences

Supplementary Result 3: Top-10 Company Feature-based Scores

Supplementary Result 4: Results Based on Linear Reputation

Supplementary Note 1: Additional Related Work

Now we discuss additional related studies in three categories, including two widely-studied talent analytics tasks (person-job-fit and career skill recommendation), along with key references related to reinforcement learning (RL) that we have adapted and enhanced with stochastic optimization techniques to address the career sequential recommendation (CSR) problem.

Person-Job Fit

Person-job fit are important components in the Human Resource Management System (HRMS)[1,2]. Efficient job fit optimizes the recruitment process, lowers the company's costs, and saves time for employees. Previous work is highly dependent on collecting the survey of the market[3–5]. Due to the competitive and dynamic nature of the job market[2,6], it often fails to capture the up-to-date information and is thus inefficient under a dynamic system. With the technological development, HRMS can collect numerous information online, which inspires companies to make a more accurate and efficient person-job fit. As AI has presented its potential in dealing with big data, many recent studies have focused on addressing the person-job fit problem using data-driven techniques. For example, Liu et al.[7] proposed a multi-source learning framework to predict job mobility and also suggest the position level, by mining the information of different career stages. By combining the hazard rate, Meng et al.[8] developed a career-path-aware neural network to recommend the next employer and the corresponding staying duration jointly. Similar work can also be found in additional references[9–11]. Paparrizos et al.[2] applied supervised machine learning methods to deal with the job fit problem by exploiting all past job transition data from both employees and employers. Unlike existing work that focuses on addressing one-step short-term career guidance, our work opens a new direction of talent analytics, aiming to offer long-term sequential recommendations for people's career life with the help of reinforcement learning.

Career Skill Recommendation

As the value of a skill depends highly on the need from the labor market[12], to remain competitive, people should continuously improve themselves to fit the market. Machine learning methods have been widely applied to guide skill recommendations. For example, Sun et al.[13] developed an enhanced neural network to assess the value of different skills in the labor market, by formulating and addressing a salary-skill value composition problem. Dave et al.[14] proposed a representation learning model by utilizing the information from job transition, job-skill, and skill co-occurrence. By constructing the tensor from large-scale recruitment data and aggregating the information from companies and skills, Wu et al.[15] applied a trend-aware approach for fine-grained skill demand analysis. Xu et al.[16] analyzed the popularity of skills by applying a Skill Popularity based Topic Model (SPTM) and rank the job skills based on their multi-faceted popularity. The state-of-the-art technique was recently developed based on the RL framework. Sun et al.[6] proposed a Skill Recommendation Deep Q-Network (SRDQN) and further confirmed its strengths in accurately measuring skill values and offer proper skill recommendations to users.. Along this line, our work also set the classical RL and deep RL as important baselines, which are widely used in many real-world sequential recommendations.

Reinforcement Learning and Stochastic Optimization

RL can be dated back to the early days of cybernetics and work in statistics, psychology, neuroscience, and computer science[17]. Inspired by the ability to work with insufficient knowledge of the neural network, deep reinforcement learning[18] (DQN) was successfully applied in the gaming field given the finite action and infinite states. Given the success of DQN, RL methods have attracted considerable attention from both academic and practical research. By continuously combating with itself, the RL model can even learn the Go game by itself without any instructions[19]. Thanks to its long-term cognitive ability and reliable performance in the Markov decision process with infinite time, RL has been applied to master chess and shogi by self-play[19,20] in strategic gaming. Further demonstrating its potential, the RL method has been widely applied in various domains of real-world applications including traffic control[21–23], traffic prediction[24], quantitative investment[25,26], etc.

On the other hand, stochastic optimization methods have been widely utilized in dealing with complex problems[27–32]. By stochastically searching in the space without any prerequisites, it can quickly locate to the global optima. By introducing the domain decomposition during the communication process, Ye et al.[27] developed an efficient parallel stochastic optimization method for mobile sequential recommendation, which is also a long-term optimization problem. Zhang et al.[33] proposed to always retain the best result among all other stochastic results and successfully applied the stochastic optimization method into the taxi-sharing problem. Guo et al.[34] analyzed the relations between the results from stochastic optimization under different initial conditions and proposed an efficient result aggregation strategy in the classification problem. In our work, we adapt the RL by combining it with the stochastic subsampling process. Our method features an important innovation in RL, enabling which to solve problems with indefinite and time-varying action space.

Supplementary Note 2: Notations and Definitions

Table S.1 summarizes important notations and their definitions in the paper.

Table S.1. Important Notations and Definitions.

Notation	Definition
P	Career path, e.g., $\{$(Google, Engineer, 14 months),(Amazon, Engineer, 16 months), ...$\}$.
C_0	The initial state (company) of an user.
C_i	The ith company in a career path.
$Pr(C_i,t)$	The staying probability in C_i at time t.
D_{C_i}	One's staying duration at C_i.
t_1	The suffering period for job hopping.
f_l	Company-related features (*Reputation*, *Popularity*, *Average Staying Duration*, and *Smooth Transfer Rate*); $l \in \{1,2,3,4\}$.
θ_l	The weight of f_l in company rating. It reflects the user preference on specific features.
β_1	Penalty term for company rating. It is applied when the job hopping company is worse than current. It reflects the improvement tendency in career path.
β_2	Penalty term for duration estimator. It shortens the duration if the model assumes that the people is not fully prepared for the job hopping company.

Supplementary Note 3: Theoretical Proofs

Here, we provide the mathematical proofs for one property, one lemma, and three propositions in the paper.

Property 1. *(**Upper Bound of Policies**) Given finite career time, if P^* is the optimal career path starting with C_0, we should have $\sum_{j\in P_i} S_j \le \sum_{j\in P^*} S_j$, for any path P_i with the same initial state and career time length.*

Proof. Assuming that there exists a path P' staring at C_0 with the same career time length and it satisfies the condition that:

$$\sum_{j\in P'} S_j > \sum_{j\in P^*} S_j. \tag{S.1}$$

Eq. (S.1) indicates that P' is the optimal one. Since we have the optimal policy π^*, according to the definition of optimal policy, we can conclude that P' is generated by π^*. Since P' and P^* both generated by π^* with the same condition, they should be equivalent to each other which contradicts the assumption. □

Lemma 1. *(**Upper Bound of Local Policy**) Given an initial company C_0, for any optimal paths generated by $\pi^*_{\mathscr{C}_{sub}}$ the optimal policy on $\mathscr{C}_{sub} \subseteq \mathscr{C}$, their accumulative rewards cannot exceed that of P^* generated by the optimal policy $\pi^*_{\mathscr{C}}$.*

Proof. Assuming that there exists a path P' generated by $\pi^*_{\mathscr{C}_{sub}}$ and it is better than the optimal path P^*. Then we can conclude that:

$$\sum_{j\in P'} S_j > \sum_{j\in P_i} S_j, \forall\, P_i \text{ starts at } C_0 \text{ with given time length.} \tag{S.2}$$

Based on Property 1, P' should also belong to $\pi^*_{\mathscr{C}}$. As P' is different from P^*, then P^* is not belong to the optimal policy $\pi^*_{\mathscr{C}}$ which contradicts the assumption. □

Proposition 1. *(**Boundary Condition**) Suppose that we define "target companies" as those appear on the globally optimal career path. The global optima can be achieved by a local policy $\pi^*_{\mathscr{C}_{sub}}$, if and only if the target companies are included in the corresponding company subset $\mathscr{C}_{sub}$.*

Proof. Necessity: To find the global optima, we must find all the target companies on it and order them properly. The path generated by $\pi^*_{\mathscr{C}_{sub}}$ will only contain the companies within $\mathscr{C}_{sub}$. If the target companies are not included in the corresponding company subset $\mathscr{C}_{sub}$, then the generated path will also not contain all the target companies which contradicts the assumption that global optima has been achieved.

Sufficiency: Assuming that all the target companies are included in $\mathscr{C}_{sub}$ and we find a path P' different from global optimal P^*. As the optimal policy is aiming at maximize the accumulative rewards, we know that the accumulative reward of P' is larger than that of P^*. Then we can conclude that P^* is not the global optimal which contradicts the assumption. □

Proposition 2. *(**Transformation Condition**) With a large number of iterations, if the quality of the optimal path cannot be further improved based on randomly generated subsets, then the current optimal path is the global optima.*

Proof. Based on Lemma 1 and Proposition 1, we know that the locally optimal policy is no better than the globally optimal policy and the globally optimal policy is also included in locally optimal policies. Given a locally optimal policy $\pi^*_{\mathscr{C}_{sub}}$, as long as it is not the globally optimal policy and given large iterations, it always has the potential to be improved as the best locally optimal policy still not be found.

As the global optima is the best local optima, if we keep on improve the current optimal path under large iterations and there is no improvement, we can know that current optimal path is the globally optimal path. □

Proposition 3. *(**Convergence Analysis**) The cool-down strategy can guarantee the convergence under the uncertain scenario.*

Proof. The decision parameter ω is defined as:

$$\omega = \begin{cases} 1, & E_2 - E_1 > 0 \\ e^{\frac{E_2 - E_1}{\Gamma^K T}}, & E_2 - E_1 \le 0 \end{cases} \tag{S.3}$$

where E_1 is the accumulative reward for the preserved optimal path, E_2 is the accumulative reward for new path, T is the temperature , K denotes the decision making times and Γ is the decay rate.

At the beginning of the iteration, as K is small, we know that

$$\Gamma^K \to \Gamma \Rightarrow \Gamma^K T \to \Gamma T. \tag{S.4}$$

If the current result is worse than the previous optimal one due to the effect of random events, then $E_2 - E_1 < 0$ and we know the decision parameter w can be estimated as:

$$\frac{E_2 - E_1}{\Gamma^K T} \to \frac{E_2 - E_1}{\Gamma T} \Rightarrow w \approx e^{\frac{E_2 - E_1}{\Gamma T}}. \tag{S.5}$$

In this case, it is possible that a uniform random variable from 0 to 1 is larger than w, so that accepting the result which indicates the high tolerance for the solution at the beginning under the random scenario.

As the iteration increases, K is gradually increasing, and when K is large enough and $0 < \Gamma < 1$, we have:

$$\Gamma^K \to 0 \Rightarrow \Gamma^K T \to 0. \tag{S.6}$$

If $E_2 - E_1 < 0$, the current result is worse than the previous one, we know that

$$\frac{E_2 - E_1}{\Gamma^K T} \to -\infty \Rightarrow w = e^{\frac{E_2 - E_1}{\Gamma^K T}} \to 0. \tag{S.7}$$

In this case, it is nearly impossible to accept a result worse than before, leading to a special case (the greedy case) of our strategy: it only accepts better solutions. As the greedy case only accepts an improvement for the previous policy, as long as the iterations are large enough, the current policy will no longer be improved and the convergence is reached according to Proposition 2. □

Supplementary Note 4: Data Format

Table S.2 listed several examples of the sequential structure in our data. Following the reference[8], our data contain 26 position types, including administrative, sales, consulting, operation, program and project management, business development, quality assurance, community and social services, arts and design, support, engineering, finance, legal, product management, healthcare services, media and communication, education, accounting, research, real estate, human resources, information technology, military and protective services, market, purchasing, and entrepreneurship.

Table S.2. Format of Available Data.

User ID	Company/Organization	Position Type	Work Duration
0s322	IBM	Support	2011/8/20 - 2014/3/15
0s322	Citibank	Consulting	2014/6/08 - 2016/2/25
0a31b	SignalFX	Engineering	2018/1/01 - Current

Supplementary Note 5: Important Data Statistics

Table S.3 reports important data statistics of the cleaned data.

Table S.3. Important Data Statistics.

Feature	Mean	Median	Maximum	Minimum	Standard Deviation
Career Length (Years)	16.2	14.2	49.9	0.0	9.5
Company Staying Duration (Years)	2.1	2.1	9.5	0.0	0.7
# of Different Companies on a Career Path	4.8	4.0	80	1	1.5

Note: The cleaned data contain 6,495,600 users from 4,281 companies and one company group. In our data there are over 500 companies that appear less than 5 times. To avoid biased measurements on their features, we consider them as a group.

Supplementary Result 1: Distribution of Average Staying Duration and Path Quality

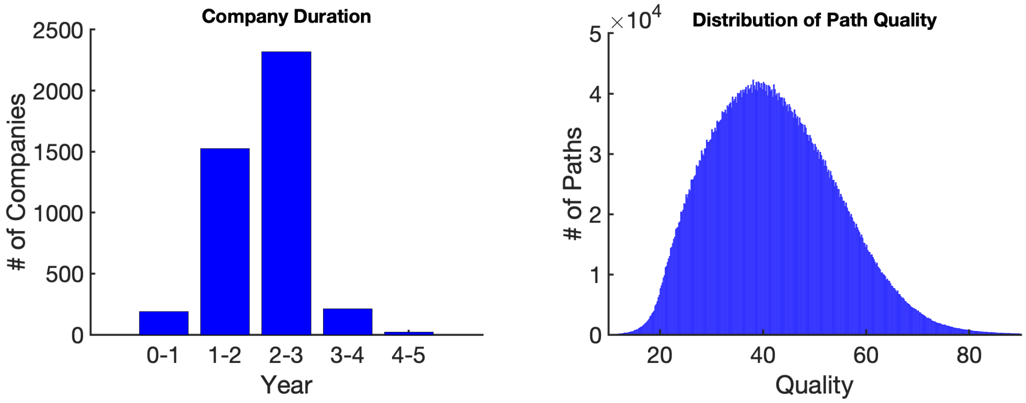

(a) Average Staying Duration

(b) Career Path Quality

Figure S.1. Real-World Distribution of Average Staying Duration and Path Quality. Both the average staying duration and career path quality are computed based on our cleaned data (see Table S.3). The figure on the left shows the distribution of the average staying duration of companies. For most companies, the average staying duration is less than three years. As shown in the figure on the right, given an equally-weighted reward function based on four company-related features (i.e., reputation, popularity, average staying duration, and smooth transfer rate), the average reward for people's career paths for the first 20 years is only 41.96 out of 100, with a standard deviation of 12.33.

Supplementary Result 2: Top-10 High-Score Recommendations under Different User Preferences

Table S.4. Top-10 High-Score Recommendations under Different User Preferences

Rank	Preferred Company/Organization Feature			
	Reputation	Popularity	Average Staying Duration	Smooth Transfer Rate
1	IBM (100)	IBM (100)	Indian Air Force (100)	IBM (100)
2	JPMorgan Chase (94.91)	Canon (95.50)	HM Forces (86.84)	JPMorgan Chase (98.12)
3	Microsoft (92.84)	Hewlett Packard (81.25)	Royal Air Force (86.46)	Lockheed Martin (94.95)
4	Pfizer (92.15)	Accenture (78.08)	Royal Navy (80.70)	Pfizer (94.70)
5	Lockheed Martin (91.40)	Ernst & Young (75.31)	U.S. Navy (79.25)	Entergy (94.68)
6	PepsiCo (91.34)	PwC (74.96)	Royal Australian Navy (76.13)	CNA (94.59)
7	Manpower Group (91.28)	Boys & Girls Clubs of America (71.81)	Royal Australian Air Force (75.74)	Aetna (94.12)
8	Walmart (90.91)	JPMorgan Chase (70.25)	British Army (72.74)	Electronic Data Systems (93.52)
9	Morgan Stanley (90.42)	Bank of America (69.90)	U.S. Air Force (70.91)	Raytheon (93.35)
10	Aig (90.28)	Banamex (65.68)	Various (69.93)	PulteGroup (93.16)

Note: This table reports the top-10 company feature-based scores, based on different user preferences on the four company features (i.e., reputation, popularity, average staying duration, and smooth transfer rate). The company feature-based scores are shown in the parentheses after the company name. The weight for the preferred company feature is set to 0.6, while others are set to 0.1. We can find that the top-10 high-score recommendations are similar to the top-10 most frequent recommendations, indicating that high-score companies are more likely to be recommended via SSRL to users according to their preferences.

Supplementary Result 3: Top-10 Company Feature-Based Scores

Table S.5. Top-10 Company Feature-Based Scores (No Specific User Preference)

	Company/Organization Feature Score				
Company/Organization	Reputation	Popularity	Average Staying Duration	Smooth Transfer Rate	Total
IBM	37.65	35.73	12.06	14.56	100
JPMorgan Chase	36.90	22.33	11.20	15.72	86.15
Microsoft	36.54	20.64	8.77	14.58	80.52
U.S. Navy	24.34	19.42	24.03	12.22	80.01
Pfizer	36.41	14.77	11.86	15.61	78.64
Lockheed Martin	36.26	11.87	12.56	15.90	76.60
PepsiCo	36.25	15.64	9.26	15.30	76.44
Manpower Group	36.23	20.12	7.75	12.17	76.27
Walmart	36.16	18.52	8.58	12.01	75.26
Morgan Stanley	36.05	13.12	10.30	14.46	73.93

Note: This table shows the top-10 scores based on the four company features (i.e., reputation, popularity, average staying duration, and smooth transfer rate) with no specific user preference. The weight for each company feature is set to 0.25. The partial score of each company feature is also reported.

Supplementary Result 4: Model Performance Based on Linear Company Reputation

To check the stability of our method under different feature value settings, we conduct a robustness test by setting company reputation as continuous scores instead of categorical values. We simulate each company's reputation by normalizing the employee size to a value between between 0 and 1. The reputation is computed as: $Reputation = \frac{Size_{C_i} - MinimumSize}{MaximumSize - MinimumSize}$. Also, we follow the experimental settings of the Scenario 1 in Figure 2, where no specific user preference is provided (i.e., the weight set of company-related features [*reputation*, *popularity*, *average staying duration*, *smooth transfer rate*] is set to [0.25, 0.25, 0.25, 0.25]. We simulate the path with 30 random initial states, and the mean values are then plotted along with their standard errors [the error bars in Figure S.2(a)]. We can find that our method outperforms all the benchmarks consistently.

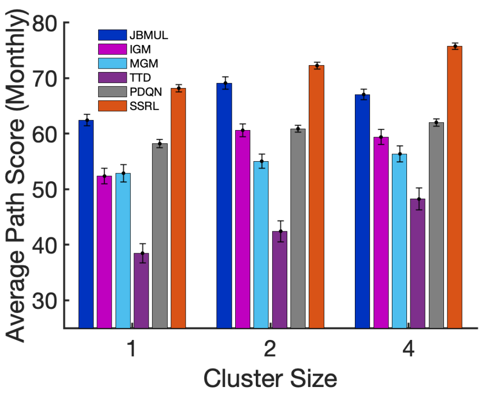

(a) Career Path Score

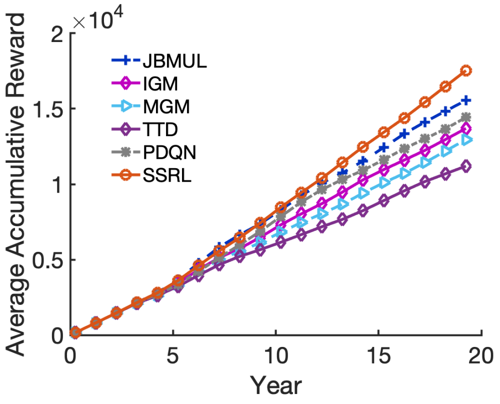

(b) Accumulative Reward

Figure S.2. Average Path Score and Accumulative Reward with Linear Company Reputation (Scenario 1). Figure S.2(a) plots the average path scores (per month); Figure S.2(b) plots the accumulative rewards of recommended career paths over a 20-year career timeline (company cluster size = 4). Here each company's reputation is represented by a continuous value between 0 and 1. All other experimental settings are the same as Scenario 1 in Figure 2.

References

1. Lee, D. H. & Brusilovsky, P. Fighting information overflow with personalized comprehensive information access: A proactive job recommender. In *Proceedings of the Third International Conference on Autonomic and Autonomous Systems*, 21–21 (2007).
2. Paparrizos, I., Cambazoglu, B. B. & Gionis, A. Machine learned job recommendation. In *Proceedings of the Fifth ACM Conference on Recommender Systems*, 325–328 (2011).
3. Murphy, K. M. & Welch, F. Occupational change and the demand for skill, 1940-1990. *American Economic Review* **83**, 122–126 (1993).
4. Maurin, E. & Thesmar, D. Changes in the functional structure of firms and the demand for skill. *Journal of Labor Economics* **22**, 639–664 (2004).
5. Outlook, O. S. First results from the survey of adult skills. *Organisation for Economic Co-Operation and Development* (2013).
6. Sun, Y., Zhuang, F., Zhu, H., He, Q. & Xiong, H. Cost-effective and interpretable job skill recommendation with deep reinforcement learning. In *Proceedings of the Web Conference 2021*, 3827–3838 (2021).
7. Liu, Y., Zhang, L., Nie, L., Yan, Y. & Rosenblum, D. S. Fortune teller: predicting your career path. In *Proceedings of the AAAI Conference on Artificial Intelligence* (2016).
8. Meng, Q., Zhu, H., Xiao, K., Zhang, L. & Xiong, H. A hierarchical career-path-aware neural network for job mobility prediction. In *Proceedings of the 25th ACM SIGKDD International Conference on Knowledge Discovery and Data Mining*, 14–24 (2019).
9. Wang, C., Zhu, H., Hao, Q., Xiao, K. & Xiong, H. Variable interval time sequence modeling for career trajectory prediction: Deep collaborative perspective. In *Proceedings of the Web Conference 2021*, 612–623 (2021).
10. Qin, C. *et al.* An enhanced neural network approach to person-job fit in talent recruitment. *ACM Transactions on Information Systems* **38**, 1–33 (2020).
11. Zhu, C. *et al.* Person-job fit: Adapting the right talent for the right job with joint representation learning. *ACM Transactions on Management Information Systems* **9**, 1–17 (2018).
12. Kokkodis, M. & Ipeirotis, P. G. Demand-aware career path recommendations: A reinforcement learning approach. *Management Science* **67**, 4362–4383 (2021).
13. Sun, Y. *et al.* Market-oriented job skill valuation with cooperative composition neural network. *Nature Communications* **12**, 1–12 (2021).
14. Dave, V. S., Zhang, B., Al Hasan, M., AlJadda, K. & Korayem, M. A combined representation learning approach for better job and skill recommendation. In *Proceedings of the 27th ACM International Conference on Information and Knowledge Management*, 1997–2005 (2018).
15. Wu, X. *et al.* Trend-aware tensor factorization for job skill demand analysis. In *IJCAI*, 3891–3897 (2019).
16. Xu, T., Zhu, H., Zhu, C., Li, P. & Xiong, H. Measuring the popularity of job skills in recruitment market: A multi-criteria approach. In *Proceedings of the AAAI Conference on Artificial Intelligence*, vol. 32 (2018).
17. Kaelbling, L. P., Littman, M. L. & Moore, A. W. Reinforcement learning: A survey. *Journal of Artificial Intelligence Research* **4**, 237–285 (1996).
18. Mnih, V. *et al.* Human-level control through deep reinforcement learning. *Nature* **518**, 529–533 (2015).
19. Silver, D. *et al.* Mastering the game of go without human knowledge. *Nature* **550**, 354–359 (2017).
20. Silver, D. *et al.* A general reinforcement learning algorithm that masters chess, shogi, and go through self-play. *Science* **362**, 1140–1144 (2018).
21. Chen, C. *et al.* Toward a thousand lights: Decentralized deep reinforcement learning for large-scale traffic signal control. In *Proceedings of the AAAI Conference on Artificial Intelligence*, vol. 34, 3414–3421 (2020).
22. Wiering, M. A. Multi-agent reinforcement learning for traffic light control. In *Proceedings of the 17th International Conference on Machine Learning*, 1151–1158 (2000).
23. Ning, Z. *et al.* Joint computing and caching in 5g-envisioned internet of vehicles: A deep reinforcement learning-based traffic control system. *IEEE Transactions on Intelligent Transportation Systems* **22**, 5201–5212 (2021).

24. Nie, L. *et al.* A reinforcement learning-based network traffic prediction mechanism in intelligent internet of things. *IEEE Transactions on Industrial Informatics* **17**, 2169–2180 (2020).

25. Wu, X. *et al.* Adaptive stock trading strategies with deep reinforcement learning methods. *Information Sciences* **538**, 142–158 (2020).

26. Liu, Y., Liu, Q., Zhao, H., Pan, Z. & Liu, C. Adaptive quantitative trading: An imitative deep reinforcement learning approach. In *Proceedings of the AAAI Conference on Artificial Intelligence*, vol. 34, 2128–2135 (2020).

27. Ye, Z. *et al.* Multi-user mobile sequential recommendation: An efficient parallel computing paradigm. In *Proceedings of the 24th ACM SIGKDD International Conference on Knowledge Discovery & Data Mining*, 2624–2633 (2018).

28. Guo, P., Ye, Z. & Xiao, K. A weighted aggregating sgd for scalable parallelization in deep learning. In *Proceedings of the 2019 IEEE International Conference on Data Mining*, 1072–1077 (2019).

29. Xiao, K. *et al.* Multi-user mobile sequential recommendation for route optimization. *ACM Transactions on Knowledge Discovery from Data* **14**, 1–28 (2020).

30. Kingma, D. P. & Ba, J. Adam: A method for stochastic optimization. *arXiv preprint arXiv:1412.6980* (2014).

31. Awasthi, P. & Sandholm, T. Online stochastic optimization in the large: Application to kidney exchange. In *Proceedings of the 21st International Joint Conference on Artificial Intelligence* (2009).

32. Badri, H., Bahreini, T., Grosu, D. & Yang, K. Energy-aware application placement in mobile edge computing: A stochastic optimization approach. *IEEE Transactions on Parallel and Distributed Systems* **31**, 909–922 (2019).

33. Zhang, L., Ye, Z., Xiao, K. & Jin, B. A parallel simulated annealing enhancement of the optimal-matching heuristic for ridesharing. In *Proceedings of the 2019 IEEE International Conference on Data Mining*, 906–915 (2019).

34. Guo, P., Ye, Z., Xiao, K. & Zhu, W. Weighted aggregating stochastic gradient descent for parallel deep learning. *IEEE Transactions on Knowledge and Data Engineering* (2020). URL https://doi.org/10.1109/TKDE.2020.3047894.
